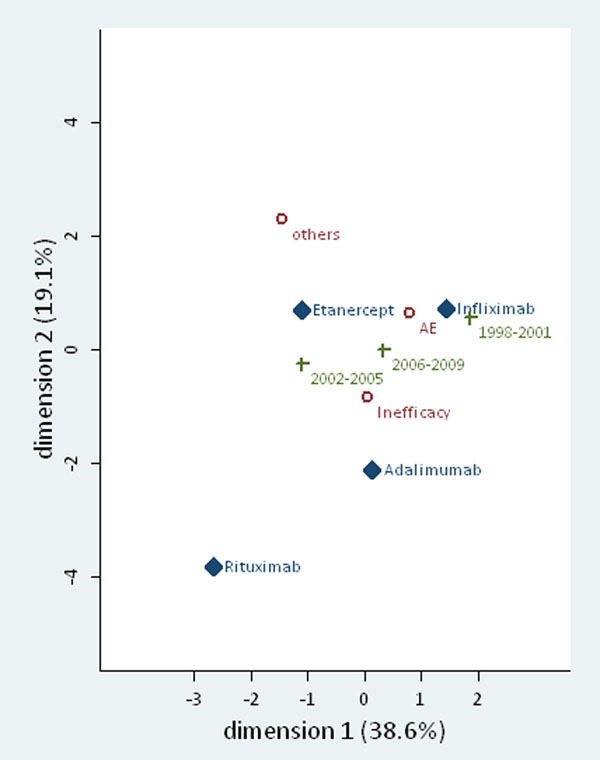# The reason to stop infliximab is clearly related to the existence of new alternatives: a correspondence analysis of biologics, reasons to stop, and time periods

**DOI:** 10.1186/1479-5876-10-S3-P28

**Published:** 2012-11-28

**Authors:** Julia Uceda, Loreto Carmona, Alejandro Muñoz, Jose L Marenco

**Affiliations:** 1 Rheumatology Dept., Valme University Hospital, Sevilla, Spain; 2 Camilo José Cela University, Madrid, Spain

## Background

The reason to stop the first biologic may vary from agent to agent and also from the earlier times to the more recent ones. Issues like the availability of newer alternatives or a better knowledge of the effectiveness and safety profile of individual biologics may influence treatment decisions, including the discontinuation of the biologic.

## Objective

To visually assess the relationship between the reason for stopping the first biologic and the period of time when the biologic was started.

## Methods

All patients with rheumatoid arthritis undergoing treatment with a biologic are registered in the clinical database of a single center with seven treating rheumatologists. The biologic used, as well as the reason to stop it is recorded, as well as all dates regarding the start and the last dose. We performed a multiple correspondence analysis with the Burt method of adjusted inertias and standard normalization with the variables: first biologic, time period (1998-2001, 2002-2005, 2006-2009), and reason to stop (adverse event, inefficacy, others). Correspondence analysis provides a means of displaying or summarizing a set of data with categorical variables in two-dimensional graphical form.

## Results

The total inertia of the exploratory analysis was 0.10. The first two dimensions added to 57% of the inertia, so that all dimensions were accounted for. The major drives of the relationship between the three variables were “infliximab” and the time period “1998-2001” (% inertia 0.146 and 0.177, respectively). The distance between other categories of the three variables was not relevant enough to make any suggestions of an association. (See Figure 1).

## Conclusions

Time has affected the decision to start infliximab, with more treatments started in the earlier times than in the later ones. The reason to stop any first biologic does not clearly relate to the time neither to the biologic itself in this single center dataset.

**Figure 1:**